# Supplementary material for: Preoperative Serum GDF-15, Endothelin-1 Levels, and Intraoperative Factors as Short-Term Operative Risks for Patients Undergoing Cardiovascular Surgery
Source: J Clin Med. 2021 May 2;10(9):1960. doi: 10.3390/jcm10091960 (PMC8125127; doi:10.3390/jcm10091960)
Supplement: Supplementary file 1 [file jcm-10-01960-s001.zip › jcm-1159906-supplementary.pdf]

Supplement Table 1. Relationships between GDF-15, ET-1, STS score, and preoperative blood data.

	**GDF-15**	**ET-1**	**STS score**
Age	**0.330 (<0.001***)**	0.114 (0.176)	**0.563 (<0.001***)**
BMI	**-0.190 (0.025*)**	-0.104 (0.217)	**-0.228 (0.010*)**
hsCRP	**0.308 (<0.001***)**	0.122 (0.147)	**0.191 (0.032*)**
Creatinine	**0.586 (<0.001***)**	**0.351 (<0.001***)**	**0.211 (0.018*)**
eGFR	**-0.664 (<0.001***)**	**-0.402 (<0.001***)**	**-0.454 (<0.001***)**
Hb	**-0.491 (<0.001***)**	**-0.253 (0.002**)**	**-0.602 (<0.001***)**
HbA1c	0.120 (0.164)	0.163 (0.054)	-0.007 (0.941)
Alb	**-0.519 (<0.001***)**	**-0.340 (<0.001***)**	**-0.497 (<0.001***)**
BNP	**0.412 (<0.001***)**	**0.630 (<0.001***)**	**0.542 (<0.001***)**
LVEF (%)	**-0.275 (0.002**)**	**-0.237 (0.006**)**	-0.143 (0.119)
ET-1	**0.514 (<0.001***)**	**-**	**0.425 (<0.001***)**
STS score	**0.545 (<0.001***)**	**0.425 (<0.001***)**	**-**

* < 0.05 ** < 0.01 *** < 0.001. BMI, body mass index; hsCRP, high-sensitive CRP; eGFR, estimated glomerular filtration rate; Hb, hemoglobin; Alb, albumin; HbA1c, hemoglobin A1c; BNP, brain natriuretic peptide; LVEF, LV ejection fraction; STS score, The Society of Thoracic Surgeons.

Supplement Table 2. Comparison of various parameters between patients with and without AKI.

	AKI (+) Total	AKI (-) Total	*P*-value	AKI (+) Male	AKI (-) Male	*P* value
Age, y	66.6 (13.1)	68.9 (13.7)	0.305	65.4 (13.7)	65.7 (14.7)	0.732
Sex number	M / F 25/4	M / F 52/ 45	-	M 25	M 52	-
BMI, kg/m^2	25.1 (5.1)	**23.3 (3.7)***	**0.034**	25.4 (5.3)	23.5 (3.5)	0.062
eGFR, ml/min/1.73 m^2	62.4 (25.3)	64.3 (20.3)	0.712	63.0 (26.8)	63.9 (22.4)	0.873
GDF15, ng/ml	2.0 (2.1)	1.3 (0.9)	0.246	2.2 (2.2)	1.3 (0.8)	0.283
hCRP, mg/l	1.4 (2.9)	0.4 (0.6)	0.106	1.4 (3.1)	0.4 (0.7)	0.303
Endothelin-1, pg/ml	1.9 (1.7)	1.2 (0.5)	0.146	2.0 (1.8)	**1.2 (0.4)***	**0.026**
Hb, g/dl	12.7 (2.1)	12.6 (1.9)	0.878	12.6 (2.2)	13.2 (1.8)	0.205
HbA1C, %	6.0 (0.8)	6.1 (0.9)	0.363	6.0 (0.8)	6.2 (1.1)	0.260
Alb, g/dl	3.8 (0.6)	4.0 (0.6)	0.350	3.8 (0.6)	4.0 (0.5)	0.247
BNP, pg/ml	354 (466)	328 (470)	0.584	393 (489)	279 (295)	0.215
STS score, %	3.5 (4.4)	2.7 (2.2)	0.698	3.5 (4.7)	1.9 (1.8)	0.063
EF, %	56.7 (12.4)	57.9 (13.0)	0.571	55.6 (12.8)	54.7 (14.0)	0.823
CPB time, h	4.2 (1.4)	**2.7 (1.1)*****	**<0.001**	4.1 (1.4)	**2.7 (1.1)*****	**<0.001**
Bleeding, ml	2501 (2404)	**1282 (965)****	**<0.001**	2530 (2571)	**1392 (1107)***	**0.015**
RCC transfusion, U	12.8 (9.9)	**6.4 (5.6)****	**0.002**	11.4 (9.7)	**5.9 (5.9)***	**0.019**
FFP transfusion, U	11.2 (9.5)	**6.4 (5.9)****	**0.003**	10.6 (10.0)	**6.6 (6.3)***	**0.046**
PC transfusion, U	20.0 (16.0)	**9.3 (11.3)****	**<0.001**	17.6 (15.4)	**8.3 (11.7)****	**0.004**
Urine volume during operation, ml	617 (402)	742 (440)	0.168	599 (422)	672 (351)	0.428
Postoperative intubation period, h	88.1 (108.8)	**15.2 (28.1)*****	**<0.001**	76.2 (110.1)	**17.5 (31.3)*****	**<0.001**
ICU period, day	5.3 (5.4)	**2.1 (3.3)*****	**<0.001**	5.1 (5.7)	**2.2 (3.0)*****	**0.000**

**P* <0.05 ***P* <0.01 ****P* <0.001 vs. patients with AKI.
